# Knowledge, attitude, and awareness of cardiopulmonary resuscitation among university enrolled medical and non-medical students of Pakistan: An online study

**DOI:** 10.12669/pjms.40.4.7933

**Published:** 2024

**Authors:** Muhammad Mudassar Bilal, Syed Bilal Shah, Tanzeel Ur Rehman, Noman Sadiq, Ghulam Rehmani Lakho

**Affiliations:** 1 Muhammad Mudassar Bilal, 4th Year MBBS Student, Mekran Medical College, Turbat, Pakistan; 2 Syed Bilal Shah, 4th Year MBBS Student, Mekran Medical College, Turbat, Pakistan; 3 Tanzeel Ur Rehman, 4th Year MBBS Student, Mekran Medical College, Turbat, Pakistan; 4 Noman Sadiq, MBBS, M.Phil. Associate Professor, Department of Physiology, Mekran Medical College, Turbat, Pakistan; 5 Ghulam Rehmani Lakho, MBBS, M.Phil. Professor Department of Physiology, Mekran Medical College, Turbat, Pakistan

**Keywords:** Cardiopulmonary Resuscitation, Awareness, Attitude, Knowledge, Pakistan

## Abstract

**Objective:**

To evaluate the awareness, attitude and knowledge of cardiopulmonary resuscitation (CPR) among university-enrolled medical and non-medical undergraduate students of Pakistan.

**Methods:**

Cross-sectional online survey-based study was conducted across institutes in Pakistan from December, 2022 to January, 2023. The study involved university-enrolled undergraduate students across the country. The structured questionnaire was disseminated via Google forms. For statistical analysis, SPSS version 20 was used to analyze the data by applying independent sample t-tests and ANOVA.

**Results:**

A total of 249 responses were received. After the exclusion of two responses, the overall awareness score of participants was found to be 2.49 ± 1.33, attitude score of 4.09 ± 1.74, and knowledge score of 3.51 ± 2.13. Female respondents, medical students, unmarried (single), private institutes, and respondents with educated parents achieved relatively higher scores. The overall difference in awareness scores among different regions of Pakistan was also significant (p <0.05). Gender, region, and parental literacy rate also showed effects on participants’ basic life support (BLS) and CPR knowledge (p <0.05).

**Conclusions:**

Overall knowledge and awareness were unsatisfactory and inadequate in university-enrolled undergraduate students, with no one getting a complete score on very basic knowledge questions. Significant differences in awareness, attitude, and knowledge among different regions, genders, and parental literacy rates were found.

## INTRODUCTION

Sudden cardiac arrest is defined as the suspension of mechanical activity of the heart with the loss of pulse, consciousness, and temporary breathing_._^1^ Out-of-hospital traumatic cardiac arrest (OHCA) causes 350,000 deaths in Europe, while in the USA, the rate is 276,000 deaths per year.^2^ A study conducted in 2018 in Karachi, Pakistan, reported the annual incidence of out-of-hospital traumatic cardiac arrest (OHCA) is calculated to be 45.7/100,000.^3^ Cardiopulmonary resuscitation (CPR) is the most crucial initial response to save a person’s life suffering from cardiac arrest, which can double the chances of survival. Delay in recognition of arrest, resuscitation, and lack of public access to an automated external defibrillator (AED) significantly reduces the chances of survival of OHCA patients, whereas OHCA has a high mortality rate.^4^ Knowledge and skill of CPR, one of the key components of Basic Life Support (BLS), has become a very crucial part of emergency response in every traumatic situation, becoming part of the healthcare academic curriculum in the USA in 1961.^5^

Studies conducted on undergraduate medical students in India indicated that the students only bother to read something apart from their curriculum and possess unsatisfactory knowledge regarding CPR.^6^ Findings of poor knowledge in medical students of pre-clinical years were reported in Saudi Arabia.^7^ Egyptian medical students also possessed generally poor awareness.^8^ A study from Malaysia reported a positive attitude and high awareness but low knowledge of BLS among students of the University of health sciences.^9^ In developing countries like Pakistan, BLS awareness is still in its initial stage.

A study conducted in Karachi, Pakistan, in 2009 also indicated that medical undergraduate students with no prior course training had poor knowledge as compared to the students with prior training.^10^ Previous studies in Pakistan showed poor knowledge of CPR among practicing doctors, dentists, and nurses among the big institutions of the largest city of Pakistan.^11^ Another study in Pakistan’s twin cities concluded that doctors had incomplete knowledge of CPR.^12^ Although most of the previous studies showed a positive attitude, the knowledge about practical skills of BLS and CPR was reported to be poor and unsatisfactory from students to serving doctors and Para-medical staff.

Responsibility for a better, secure future relies on today’s students, who will become the first-line respondents in response to tragedies both in medical and non-medical fields. Satisfactory awareness and accurate, reliable knowledge of performing CPR are important not only for Health professionals but also for the general masses. Very few studies are being done on medical undergraduates within Pakistan, and no previous research has been conducted comparing the overall awareness, attitude, and knowledge among different regions of Pakistan. There is no published data available so far on awareness and knowledge of BLS and/or CPR from the province of Baluchistan. The international studies have reported that medical students have more knowledge than the non-medical students but in Pakistan such comparison has never been reported before and our study aims to fill the gap. Our study aims to evaluate the awareness, attitude, and knowledge of CPR among university-enrolled undergraduate students in Pakistan.

## METHODS

A cross-sectional online survey-based study was conducted across institutes in Pakistan. The study was conducted from December, 2022 to January, 2023. The study was conducted by the students of Mekran Medical College Turbat Baluchistan under the supervision of Department of Physiology. It was conducted among medical and non-medical undergraduate students currently enrolled in different university programs from Baluchistan, Khyber Pakhtunkhwa (KPK), Punjab, and Sindh; the students from Islamabad capital territory were considered within the province of Punjab. The students enrolled in the bachelor program or equivalents were included in the study while students of postgraduate programs were excluded.

### Ethical Approval

It was obtained from the ethical review committee under Ref No. MMC/ERC/3-2022 on 8^th^ December 2022.

In order to evaluate the awareness, attitude, and basic practical knowledge about CPR, a simple and structured questionnaire was developed and applied. The questionnaire comprised demographical questions, four questions for assessing awareness, and seven questions about the attitude of students towards BLS, while 10 questions were related to fundamental practical knowledge about CPR according to American Heart Association guidelines 2020. The demographical questions included age, gender, field of study (medical or non-medical), status of institute (public sector or private sector), literacy of parents, marital status of respondents, and regions of country.

Informed consent was taken from every respondent. A pilot study was conducted, and 30 responses were collected to evaluate the desired responses, and the questionnaire was re-structured, to minimize the confusion and ambiguity in questions. The finalized online questionnaire was distributed as a Google-form among the students through social media groups of different institutes of all four provinces using WhatsApp. The undergraduate student population throughout the universities of Pakistan for 2022-23 was considered unlimited and sample size was calculated to be 384 using Software OpenEpi at confidence level of 95% and precision of 5%. The non-probability convenient sampling technique was adopted. SPSS version 20 was used to analyze the results. Independent sample t-tests and ANOVA tests were applied to calculate the results. p<0.05 was considered significant.

## RESULTS

A total of 249 responses were received, and two incomplete responses were excluded. Among the participants 148 (59.9%) were males, 224 (90.7%) were 18-24 years old, and 241 (97.6%) were single. A total of 116 (47.0%) respondents were currently enrolled in public sector institutions and 181 (73.3%) were medical and allied students. The majority of the respondents, 131 (53.0%), were from Baluchistan, 45 (18.2%) from Sindh, and 44 (17.8%) from Punjab, while 27 (10.9%) were from KPK. 207 (83.8%) of respondents were children of educated parents.

Overall, 149 (60.3%) respondents reported knowing nothing about BLS; however, 151 (61.1%) knew how to perform CPR. Abbreviations of BLS and CPR were correctly answered by 185 (74.9%) and 182 (73.7%) participants, respectively. About 16.6% of participants had performed CPR, while 33.2% reported having witnessed CPR being performed in some way in their life. None of the participants scored ten on knowledge questions. Participants’ overall mean awareness score was found to be 2.49 ± 1.33, attitude score of 4.09 ± 1.74, and knowledge score of 3.51 ± 2.13.

Respondents aged 18-24 years scored higher mean scores on awareness and knowledge, while those < 18 years showed relatively higher attitudes. Female participants gained higher mean scores than males in all perspectives. Medical students obtained higher scores of awareness, attitude, and knowledge than non-medical students (p <0.05). Participants with educated parents showed significantly high scores (p<0.05). The difference in scores was observed in awareness and knowledge among different regions (p <0.05), although all regions showed a positive attitude towards CPR. Participants from private institutions and of single marital status showed relatively high mean scores (Table-I & Table-II). Individual questions and response scores are shown in (Table-III).

**Table-I:** Comparison of mean scores of awareness, attitude, and knowledge of CPR among gender, field of study, institutional status, parental literacy and marital status (N= 247).

Variables	Mean ±Std. deviation		Mean difference	t-value	p-value
Gender	Male	Female			
Awareness	2.24 ±1.35	2.87 ±1.22	-0.63	-3.71	.000*
Attitude	3.96 ±1.79	4.30 ±1.66	-0.34	-1.52	.129
Knowledge	3.17 ±2.09	4.04 ±2.09	-0.87	-3.21	.002*
Field of study	Medical	Non-Medical			
Awareness	2.88 ±1.16	1.44 ±1.20	1.44	8.55	.000*
Attitude	4.32 ±1.75	3.48 ±1.59	0.84	3.4	.001*
Knowledge	4.10 ±1.91	1.92 ±1.89	2.18	7.94	.000*
Status of institutions	Public Sector	Private Sector			
Awareness	2.28 ±1.35	2.73 ±1.27	-0.45	-2.69	.008*
Attitude	3.99 ±1.74	4.22 ±1.74	-0.22	-1	.316
Knowledge	3.33 ±2.03	3.73 ±2.23	-0.4	-1.49	.137
Literacy of Parents	Educated	Not-educated			
Awareness	2.59 ±1.33	2.00 ±1.24	0.59	2.59	.010*
Attitude	4.22 ±1.74	3.45 ±1.65	0.77	2.59	.010*
Knowledge	3.71 ±2.10	2.55 ±2.05	1.16	3.2	.002*
Marital Status	Single	Married			
Awareness	2.52 ±1.32	1.33 ±1.51	1.19	2.18	.030*
Attitude	4.10 ±1.74	4.00 ±2.19	0.1	0.14	.890
Knowledge	3.55 ±2.12	2.33 ±2.66	1.21	1.38	.169

Independent Sample t-test was used,

* = p-value < 0.05.

**Table-II:** Comparison of mean scores of awareness, attitude, and knowledge of CPR among various age and regional groups (N= 247).

	Mean ±Std. deviation				F-value	p-value
Age	<18	18-24	25-30			
Awareness	1.55 ±1.29	2.55 ±1.30	2.33 ±1.61		3.126	.046*
Attitude	4.18 ±1.94	4.12 ±1.74	3.67 ±1.78		0.39	.677
Knowledge	2.45 ±1.81	3.59 ±2.13	3.08 ±2.19		1.771	.172
Regions	Balochstan	KPK	Punjab	Sindh		
Awareness	2.18 ±1.36	3.22 ±0.80	2.98 ±1.17	2.51 ±1.38	7.714	0.00*
Attitude	3.86 ±1.73	4.11 ±1.58	4.50 ±1.93	4.38 ±1.63	1.986	0.12
Knowledge	3.13 ±2.11	4.44 ±1.97	4.39 ±1.85	3.24 ±2.21	6.199	0.00*

ANOVA was used,

* = p-value < 0.05.

**Table-III:** Awareness, attitude and knowledge of cardiopulmonary resuscitation of study participants (N=247).

Questions	Responses			

Awareness	Yes		No	

	n	%	n	%
Do you know anything about BLS?	98	39.7	149	60.3
Do you know how to perform CPR?	151	61.1	96	38.9
	
	*Correct*		*Incorrect*	

	*n*	*%*	*n*	*%*
	
What does BLS mean?	185	74.9	62	25.1
What does CPR stand for?	182	73.7	65	26.3
	
*Attitude*	*Yes*		*No*	

	*n*	*%*	*n*	*%*
	
Have you ever taken any training on how to perform CPR?	85	34.4	162	65.6
Did you ever try to learn about CPR by Yourself?	128	51.8	119	48.2
Would you feel comfortable doing CPR on an opposite-gender person?	195	78.9	52	21.1
Have your current Institute held any BLS awareness/ training session?	92	37.2	155	62.8
	
	*Confident*		*Not Confident*	

	*n*	*%*	*n*	*%*
	
How confident do you feel about performing CPR on someone if you witness any accident/trauma?	147	59.5	100	40.5
How confident do you feel to perform CPR on your near and dear if there’s an emergency?	140	56.7	107	43.3
	
	*Important*		*Not important*	

	*n*	*%*	*n*	*%*
	
What is your opinion about having training sessions about BLS?	227	91.9	20	8.1
	
Knowledge	*Correct*		*Incorrect*	

	*n*	*%*	*n*	*%*
	
CPR is performed to secure what 3 key life components?	181	73.3	66	26.7
What is the recommended sequence of performing CPR?	80	32.4	167	77.6
In order to perform CPR, what is the most efficient position of the patient?	149	60.3	98	39.7
What is the proper rate of chest compression: artificial breathing during CPR per cycle?	91	36.8	156	73.2
On which area of the chest, the chest compression should be applied?	129	52.2	118	47.8
What must be the rate of the chest compressions per minute?	44	17.8	203	82.2
How many hands should be used in adults: Child?	104	42.1	143	57.9
How much force must be applied during chest compression in adults?	70	28.3	177	71.7
How much force must be applied during chest compression in a child?	77	31.2	170	68.8
How long CPR should be performed?	49	19.8	198	80.2

## DISCUSSIONS

Without any opposing debate, CPR is the most crucial step in emergency conditions of sudden cardiac arrest. CPR increases the survival rate two to three times for a person in traumatic situations, but unfortunately in developing countries, awareness and knowledge regarding BLS and/or CPR are not satisfactory.^5^ Trainings and hands-on workshop regarding basic life support bear promising results with significant accuracy and retention over time in children.^13^ Our study was conducted to find the overall awareness, attitude and knowledge of CPR among the undergraduate students. We have found that the overall fundamental knowledge about the CPR was significantly unsatisfactory among university undergraduates with poor awareness. However, the study found out positive attitude of students concerning CPR training sessions and learning opportunities. The field of study, gender, regions, and parental literacy showed significant association with the awareness, attitude, and knowledge of CPR.

Our current study showed that the medical students possess significantly higher levels of awareness and attitude regarding CPR than non-medical students. The knowledge levels among medical students, despite being higher than non-medical students, are not satisfactory as none of the participants answered all questions correctly. In comparison between medical and the non-medical students, the results indicated poor knowledge and awareness among the non-medical students compared to the students of medical and allied sciences. There are no previous studies conducted in the region of Baluchistan however the study from Pakistan reported the lack of knowledge and skill sets among the medical and non-medical personnel in Karachi.^14^ Another study from Karachi reported that the medical students lagged behind in cognitive awareness with very low skill level; however the study reported very positive attitude concerning the importance of BLS.^10^ The working health care professionals from Karachi and twins’ cities Rawalpindi and Islamabad also reported to have insufficient knowledge regarding CPR during the course of their practical life.^11^,^12^ The study from India found inadequate awareness and knowledge in undergraduate students.^6^ A study in Malaysia also reported that health care students have low BLS knowledge scores but high attitude and awareness scores.^9^ A study from Iran also reported a higher attitude but poor scores of awareness among university students.^15^ Similarly, the Saudi Arabia also reported the poor and insufficient among the university undergraduates especially the non-medical students.^16^-^18^ Studies from India and Saudi Arabia reported unsatisfactory incomplete knowledge scores of CPR specifically among medical students.^4^,^7^

Our study indicated that females tend to have comparatively higher knowledge, awareness, and attitude regarding CPR. The association between gender and CPR awareness, attitude and knowledge has never been reported throughout the country. However, our findings are consistent with the studies carried out in Saudi Arabia reporting that female students showed comparatively higher awareness and knowledge.^16^,^19^

In our study, the Participants from the region of Punjab, and KPK scored highest on the overall mean scores, followed by Sindh. Participants from Baluchistan had comparatively the lowest overall mean scores on awareness, attitude and knowledge than other regions. Although no previous study has reported the comparison of awareness, attitude, and knowledge of CPR among different regions of Pakistan, a study did report a significantly poor awareness and knowledge about Chikungunya in Balochistan compared to other regions of the country.^20^ This poor awareness, attitude, and knowledge regarding CPR could be due the result of institutional negligence, limited access to resources, infrequent exposure to traumatic situations, lack of facilities, or decline in the knowledge of students due to time gap after the training session.^21^

The results indicated the inadequate attention given to overall BLS and/or CPR education by institutions throughout the Pakistan as one third of the respondents reported to have no prior session on BLS and/or CPR at all. Although the hands-on training sessions are found to enhance the knowledge retention and accuracy of BLS^13^, but the students tend to forget the CPR learning from workshops over the course of few months due to non-continuous trainings.^22^ The lack of sufficient BLS and/or CPR training was reported to be one of the factor for the poor knowledge of CPR even among the doctors and other health care workers of Pakistan.^11^,^12^ The doctors with practice gaps performing CPR in practical life also showed insufficient knowledge of CPR.^23^ The lack of proper hands-on training may be one of the factors resulting as the poor knowledge scores among the students in our study but the determination of the exact causes of this insufficient knowledge could be multifactorial. There is a dire need of hands-on BLS and/or CPR training workshops to be included in universities’ curriculum.

The relationship between parental literacy and CPR awareness was not reported previously, however the results indicate this potential association. Respondents with educated parental backgrounds obtained higher scores. The effect of parental literacy on respondents’ awareness and knowledge of CPR may result from the direct inclusion of CPR as part of child’s education or perhaps the mere observation of a parent performing CPR in front of respondents. A previous study indicated that the health and related awareness of children is significantly affected by parental education level but the effects of low parental education and awareness during early adulthood are reported to play an insignificant role.^24^,^25^ However, the results of our study showed significant association between parental literacy and CPR awareness, attitude and knowledge among respondents. We were not able to determine the possible cause of this association and the effects of parental education on CPR awareness and knowledge levels in offspring’s adulthood life and needs to be further investigated.

Our study addressed the situation of awareness, attitude, and knowledge of BLS and/or CPR in the region of Balochis tan as well as the comparison with other regions of the country and overall condition of CPR awareness, attitude, and knowledge among the university undergraduate throughout Pakistan.

### Limitations

The study was conducted online therefore only the students with accessible internet could participate. The limited time duration of the study coincided with the annual exam schedules of the students from KPK, Sindh, and Punjab, therefore majority of the participants belongs from the region Baluchistan. Despite the repeated reminders the responses did not increase. However, the study has representation from all four provinces. However, we could not assess the practical skills of students.

## CONCLUSION

Overall knowledge and awareness were found to be unsatisfactory and inadequate in university-enrolled non-medical students compared to medical students. In addition, none of the respondents achieved a complete score on very basic knowledge-related questions. The study also indicated significant differences in awareness and knowledge among different regions, genders, and parental literacy rates.

### Recommendations

- It is recommended to conduct the study on a larger scale.
- Further studies are required to evaluate and determine the underlying causes and obstacles to BLS and CPR education.
- Institutions are requested to include hands-on CPR training sessions in their academic curriculum.

### Authors Contribution:

**MMB, SBS** & **TR:** Contributed in conceptualization of study, collected data & write-up.

**NS** & **GRL:** Contributed in conceptualization of study, data analysis, critical appraisal & final approval.

**MMB:** Is responsible for the integrity and accuracy of the study.
